# Construction and application for QTL analysis of a Restriction Site Associated DNA (RAD) linkage map in barley

**DOI:** 10.1186/1471-2164-12-4

**Published:** 2011-01-04

**Authors:** Yada Chutimanitsakun, Rick W Nipper, Alfonso Cuesta-Marcos, Luis Cistué, Ann Corey, Tanya Filichkina, Eric A Johnson, Patrick M Hayes

**Affiliations:** 1 Crop and Soil Science Department, Oregon State University, Corvallis, Oregon, USA; 2 Floragenex, Inc., Eugene, Oregon, USA; 3 Departamento de Genética y Producción Vegetal. Estación Experimental de Aula Dei. Consejo Superior de Investigaciones Científicas. Zaragoza, Spain

## Abstract

**Background:**

Linkage maps are an integral resource for dissection of complex genetic traits in plant and animal species. Canonical map construction follows a well-established workflow: an initial discovery phase where genetic markers are mined from a small pool of individuals, followed by genotyping of selected mapping populations using sets of marker panels. A newly developed sequence-based marker technology, Restriction site Associated DNA (RAD), enables synchronous single nucleotide polymorphism (SNP) marker discovery and genotyping using massively parallel sequencing. The objective of this research was to assess the utility of RAD markers for linkage map construction, employing barley as a model system. Using the published high density EST-based SNP map in the Oregon Wolfe Barley (OWB) mapping population as a reference, we created a RAD map using a limited set of prior markers to establish linakge group identity, integrated the RAD and prior data, and used both maps for detection of quantitative trait loci (QTL).

**Results:**

Using the RAD protocol in tandem with the Illumina sequence by synthesis platform, a total of 530 SNP markers were identified from initial scans of the OWB parental inbred lines - the "dominant" and "recessive" marker stocks - and scored in a 93 member doubled haploid (DH) mapping population. RAD sequence data from the structured population was converted into allele genotypes from which a genetic map was constructed. The assembled RAD-only map consists of 445 markers with an average interval length of 5 cM, while an integrated map includes 463 RAD loci and 2383 prior markers. Sequenced RAD markers are distributed across all seven chromosomes, with polymorphic loci emanating from both coding and noncoding regions in the *Hordeum *genome. Total map lengths are comparable and the order of common markers is identical in both maps. The same large-effect QTL for reproductive fitness traits were detected with both maps and the majority of these QTL were coincident with a dwarfing gene (*ZEO) *and the *VRS1 *gene, which determines the two-row and six-row germplasm groups of barley.

**Conclusions:**

We demonstrate how sequenced RAD markers can be leveraged to produce high quality linkage maps for detection of single gene loci and QTLs. By combining SNP discovery and genotyping into parallel sequencing events, RAD markers should be a useful molecular breeding tool for a range of crop species. Expected improvements in cost and throughput of second and third-generation sequencing technologies will enable more powerful applications of the sequenced RAD marker system, including improvements in *de novo *genome assembly, development of ultra-high density genetic maps and association mapping.

## Background

Plant breeders and geneticists have benefited from the availability of tools for the rapid and cost-effective development of molecular marker-based linkage maps. As predicted by Tanksley et al. [1], linkage maps have proven to be useful for discovering, dissecting and manipulating the genes that determine simple and complex traits in crop plants. Barley (*Hordeum vulgare*) is a model for plant breeding and genetics because it is diploid (2n = 2x = 14) and has a long history of genetics research. Over the past decade, increasingly dense maps of the barley genome have been constructed using multiple populations and many types of molecular markers [2]. Most recently, Szűcs et al. [3] reported an integrated 2383-locus linkage map developed in the Oregon Wolfe Barley (OWB) mapping population based on representative early generation markers (e.g. morphological loci, RFLPs, and SSRs) and single nucleotide polymorphisms (SNPs).

SNP markers have become increasingly important tools for molecular genetic analysis, as single base-pair changes are the most abundant small-scale genetic variation present between related sequences of DNA [4]. To date, most SNP development efforts in larger, more complex genomes such as barley have focused on "complexity reduction" techniques that aim to sequence a fraction of the genome, such as that represented in EST collections. Once a panel of markers is established from initial SNP discovery, samples from a selected population are then genotyped using oligo-extension or array-based platforms [5]. Both these strategies were used for construction of the current barley SNP-based maps [3,6,7].

The emergence of massively-parallel, next-generation sequencing (NGS) platforms capable of producing millions of short (50-100 bp) DNA sequence reads has reduced the costs of DNA sequencing and offers the tantalizing possibility of making direct, genotyping-by-sequencing (GBS) practical (Reviewed in [8]). Recently, Huang and colleagues [9] have elegantly demonstrated how genotyping using NGS data can facilitate the rapid development of linkage maps in domesticated rice, *Oryza sativa*. Despite the attractiveness of this approach and availability of next-generation sequencing platforms, at present, GBS methods retain significant limitations. First, current protocols for synthesis of DNA fragment libraries compatible with high-throughput sequencing platforms are laborious, costly and would be impractical for production efforts involving hundreds of samples [10]. Second, sequence-based genotyping is restricted to those species with available, high-quality, pseudomolecule-sized genome assemblies [9]. While many key economic and scientifically meritorious species will undoubtedly be sequenced as a direct result of the ongoing revolution in NGS technologies, what is required are marker platforms that can provide GBS independent of the status of an assembled genome.

Restriction-site Associated DNA (RAD) markers detect genetic variation adjacent to restriction enzyme cleavage sites across a target genome [11]. The first iteration of RAD markers facilitated cloning of mutants isolated from genetic screens in classic model systems [12,13]. More recent efforts have focused on adapting the RAD technique for use in NGS platforms, specifically the Illumina sequencing-by-synthesis method, to enable individual sequence based genotyping of samples [14]. The sequenced RAD marker system enjoys two favourable characteristics for high-throughput GBS. As previously mentioned, the RAD method uses restriction enzymes as a complexity reduction strategy to reduce the sequenced portion of the genome anywhere from 0.01% to 10% [15]. Furthermore, RAD protocols facilitate the creation of highly multiplexed NGS sequencing formulations containing many tens of samples in a single library, thereby reducing library preparation costs [14]. While previously published RAD studies have explored NGS of limited numbers of individuals or bulked genotyping of pooled populations, the objective of this research was to determine the feasibility of constructing a RAD marker genetic map in barley. We used the OWB population as a mapping resource in order to directly compare RAD and EST-based SNP maps and to assess the quality and utility of a linkage map built with the two types of data.

## Results & Discussion

### Genome Analyzer sequence results, SNP Discovery and DH Genotyping

A total of 2,010,583 36-bp sequence reads were obtained for the OWB dominant and recessive inbred genetic stocks (parents of the OWB mapping population), while 27,704,592 sequence reads were obtained for the 93 member DH mapping population (Additional file 1: Table S1). Illumina sequences from the OWB parental lines were first used for identification of SNPs. Putative alleles were mined from the sequence data using several custom PerlScripts and filtering procedures. First, raw 36-bp Illumina sequence reads were partitioned into discrete files using a 5' multiplex identifier (MID) corresponding to each OWB sample and the restriction enzyme site *SbfI *(TGCAGG). Segregated data from each line was then collapsed into putative RAD sequence clusters comprised of a minimum of eight (8×) redundant sequence reads per locus. Sequences not attaining the 8× sequence coverage threshold were excluded from further analysis, as were putative high-copy RAD sequences where the number of sequence occurrences in each cluster was greater than 500 (500×). Homologous RAD clusters from the dominant and recessive lines were then compared using a custom k-mer matching algorithm permitting exact sequence matches (monomorphic loci), single mismatch (one SNP per read) and two nucleotide mismatches (two SNPs per read) per 28 bp sequence. An initial panel of 530 SNPs with fixed genotypes in both parents were identified using these criteria and alleles for each marker were assigned to their respective parental donor (Additional file 2: Table S2).

The putative 530 SNP marker panel was then used to score RAD sequences obtained from each of the DH individuals. As alleles are fixed within each member of the doubled haploid OWB population, we posited sequence genotypes could be accurately determined at low sequence coverage (<5×) [16]. To further minimize genotyping miscalls due to possible sequencing errors, a minimum of two independent sequence reads were required over any locus to assign any SNP genotype. Putative genotypes developed for individual samples were converted into JoinMap 4 [17] compatible format using custom PerlScript. Loci lacking sufficient sequence coverage or with conflicting genotype data were coded as missing data.

### Linkage map

We used the following criteria to assess the quality of the RAD markers for linkage map construction. First, with the RAD-only map we considered the (i) total number of loci detected, (ii) the percentage of polymorphic loci, (iii) the number of missing allele calls for polymorphic loci, (iv) the percentage of codominant loci, (v) segregation distortion, (vi) the number of significant singletons (vii) linkage map length, and (viii) the number, location, interaction and effect of significant QTL. For purposes of comparison, we used the map reported by Szűcs et al. [3]. Subsequently, we added the RAD data to the 2383 locus map and assessed criteria v - vii, above. For criteria viii, however, we used a skeleton map, as described in the Methods. First, we will present results in terms of criteria i - vii; the QTL results will be presented separately.

**Figure 1:**
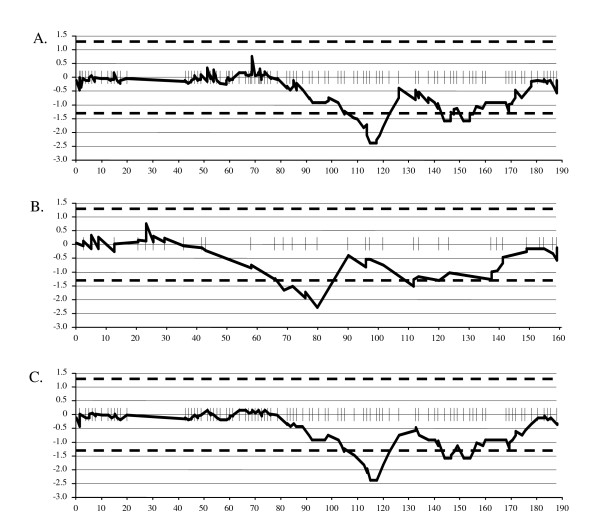
**Segregation distortion on chromosome 2H linkage maps in the Oregon Wolfe Barley mapping population**. The results of mapping with two different data sets are shown in A) the OWB-2383 map + 463 RAD loci, B) the 436 RAD and morphological marker loci and C) the OWB-2383 map. The X axis represents map distance in cM and the Y axis represent *-log *of the χ^2 ^*p*-value for segregation distortion. A positive value means distortion in favor of OWB-D whereas a negative value means distortion in favor of OWB-R. Dashed lines represent significance thresholds at 0.05. Marker positions are represented as perpendicular lines to the X axis.

Of approximately 10,000 RAD sequence clusters interrogated, 530 loci (5.3%) were classified as codominant markers where two distinct alleles were explicitly observed between the OWB parents. A number of dominant-style markers, which are sequences present in one parent but not the other, were also observed within the data but were not used for map construction as dominant markers have reduced genotyping quality. Of the codominant RAD marker class, 67 (13%) were excluded from further analysis due to missing data (≥ 15% missing data points). This left 463 (4.6% of the total) RAD loci, plus the nine morphological markers, for map construction. Twenty-seven RAD markers remained unlinked at LOD 5.0 and the remaining 436 formed seven linkage groups, together with the nine morphological markers. Based on visual assessment of locus orders, there were 22 loci showing apparent double crossover events. Of these, 23 singletons data points were re-coded as missing data for 20 loci where these occurred, except for two loci where distances between flanking markers were large enough to expect recombination. The final map is composed of 436 RAD and nine morphological markers. The total length of the RAD map is 1260 cM. Excluding co-segregating markers, the average marker density is 5 cM (Additional file 3: Figure S1). Significant segregation distortion was observed on chromosomes 2H, 3H, 6H, and 7H (Figure 1). On chromosomes 2H and 3H the segregation distortion was in favor of the OWB recessive parent allele and on chromosomes 6H and 7H it was in favor of the OWB dominant parent allele. The lengths (in Haldane cM) for each linkage group are shown in Table 1.

**Table 1:** Summary of chromosome length in three linkage maps.

Linkage map	Chromosome							Total
		
	1H	2H	3H	4H	5H	6H	7H	
OWB-2383*	158	188	208	127	238	163	206	1288
OWB-2383 + 463RAD	158	188	208	127	238	163	204	1286
RAD only	175	158	228	123	226	134	216	1260

*The OWB-2383 map was reported by Szűcs et al. [3].

Lengths (in Haldane cM) for linkage groups corresponding to barley chromosomes 1H - 7H of the Oregon Wolfe Barley mapping population.

For construction of the RAD + 2383 locus map, the same 463 RADs selected initially for the RAD-only map were added to the 2383-locus data set reported by Szűcs et al. [3]. The 23 singletons were replaced by missing values. The combined map therefore consists of 2846 loci and has a total length of 1286 cM (Table 1). Marker orders for the non-RAD markers are consistent between the 2383 and 2846-locus maps. Seventy-eight percent (359) of the RAD markers co-segregate with one or more of the previous markers. There were examples of gap-filling: for example, FGX_OWB00091, mapped to a 17 cM gap on chromosome 7H in the Szűcs et al. [3] and incorporation of this marker reduced the distance between the two flanking markers to 10 cM. Segregation distortion was observed at the same positions as in the RAD-only map (Figure 1). The lengths (in Haldane cM) for each linkage group are shown in Table 1. The same lines identified as identical with the RAD-only data (see Methods, Plant material) were confirmed as identical using the 2383 data points reported by Szűcs et al. [3].

Although a significant number of RAD loci were eliminated based on lack of polymorphism and missing sequence data, the genome scan uncovered over 400 high quality loci that were available for map construction. By way of comparison, there are 722 DArT loci on the Szűcs et al. OWB map [3], out of the 1,500 loci that were genotyped. The RAD loci are codominant whereas DArTs are dominant markers [18]. In the case of dominant markers, missing data due to error vs. allele absence cannot be distinguished, and this leads to a higher frequency of apparent singletons in map construction. The high quality of the RAD data is further confirmed by the comparable linkage map lengths for the RAD only, RAD + prior marker, and Szűcs et al. OWB map [3](Table 1). Segregation distortion was observed in all maps at the equivalent positions confirming that this was due to non-random distribution of alleles to haploid progeny and not to scoring errors. The pronounced segregation distortion on 2H is attributable to the *ZEO *locus, with selection against the "dwarfing" alleles of the dominant parent.

The presence of duplicate sets of lines in the OWB population provides an additional test for data quality. The members of each set were not identified as identical in previous iterations of the map (e.g. Costa et al. [19]) due to differences at loci that have been progressively removed from the data set based on quality control criteria. The lines within each subset are identical for the Illumina SNPs and all other loci included in the Szűcs et al. OWB map [3]. That the lines within each set are also identical for all RADs confirms the repeatability of the RAD genotyping assay and that the lines are identical. The most likely explanation for the presence of these identical sets of lines in the population is that multiple haploids were inadvertently advanced from callus regenerated from a single embryo. Removal of the sets of identical lines reduces the mapping population size from 93 to 82. There are no differences in locus order between the n = 93 and n = 82 maps and map lengths are comparable [20].

### EST and genome mapping of RAD sequence markers

The RAD technique develops sequence from regions adjacent to restriction endonuclease digestion sites in a target genome [14]. To establish if sequence-based RAD markers from the OWB genetic map would anchor to existing *Hordeum *genomic resources, we used the short-read aligner Bowtie to map RAD sequences onto a barley gene index [21,22]. Using this database, we successfully identified unique alignment positions for 51 of 436 sequenced RAD loci (11.0%). An additional 22 RAD loci (4.7%) mapped to multiple positions in the gene index. A list of summary alignments for all RAD markers in this database can be found in additional file 4: Table S4. Although the gene index contains approximately 54 Mb of putative coding sequence distributed across 80,723 tentative assemblies, this database spans only a small fraction (~0.1%) of the 5.0 Gb barley haploid genome. As Ty3 and Copia retrotransposon families are believed to inhabit a large portion of the barley genome, we postulated some percentage of RAD sequences might originate from repetitive-class sequences [23]. However, several attempts to align the 463 RAD sequence loci to the 1.3 Mb TIGR *Hordeum *repeat database under a variety of thresholds did not reveal any successful alignments. A larger percentage of RAD sequences could be positioned on candidate genes than would expected by random sampling, suggesting that RAD markers are significantly enriched in the gene space. The absence of any alignments to known repetitive sequences also hints that RAD markers are clustered within recombinatorially active regions of the genome.

### Comparative Genome Analysis

**Figure 2:**
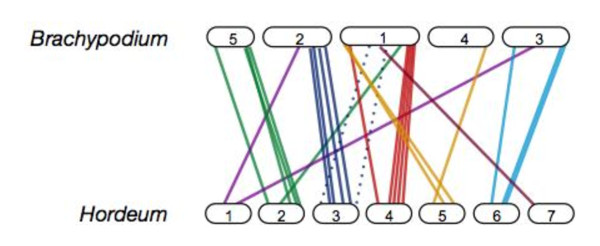
**Macro-scale syntenic relationships between barley and *Brachypodium *revealed with sequenced RAD markers**. RAD sequences anchored by linkage analysis are distributed across the seven *Hordeum *linkage groups. Alignments to orthologous sequence loci in *Brachypodium *are shown. Solid lines denote relationships supported by EST sequence comparison. Two dashed lines indicate sequence alignments that do not coincide with expected chromosomal relationships.

To examine if assembled grass genomes would serve to anchor other RAD markers, we aligned polymorphic sequences to the 430 Mb *Oryza sativa *and 300 Mb *Brachypodium distachyon *genomes using a modified CIP/CALC method [24-26]. Bowtie alignment results using relaxed parameters indicate that only 16 and 24 of the 463 OWB RAD sequences mapped to either the rice or *Brachypodium *chromosome assemblies, respectively. Despite the small number of orthologous RAD sequences and the short Illumina read of 28 bp, alignments of RAD markers ordered by the genetic map against the finished *Brachypodium *genome (Figure 2 and additional file 5: Figure S5) agree with macro-scale syntenic relationships established by previous efforts [25]. Although this study has relatively few sequence loci available for comparison, our findings suggest that a denser RAD marker scan, using a more frequently cutting restriction enzyme would interrogate more genome sequence and interrogate more sequence for comparative analyses.

Overall, we were able to assign 74 of 463 RAD sequence loci (15.9%) to at least one of the three sequence references, leaving the genomic origin of the remaining barley RAD tags (389 loci, 84.1%) unknown. We postulate the large numbers of RAD sequences placed on the OWB linkage without homology or orthology to known sequences are a result of two factors. First, the lack of a contiguous barley genome, which would allow us to explicitly determine the location of all RAD sequences, restricts our analysis to the small fraction of the haploid genome that has been sequenced. Second, despite established syntenic relationships between the *Oryza, Hordeum *and *Brachypodium *genomes, the inefficient mapping of barley sequenced RAD markers across species is likely a result of the majority of RAD loci emanating from areas of the barley genome which have significantly diverged at the nucleotide level since the speciation of the Poaceae [27,28].

A cohesive explanation for the results observed in the genetic map and comparative genome analysis is that the majority of RAD loci are linked with, but lie outside gene sequences. In this study, although only 11.0% of RAD sequences align to known barley genes, we report 78% of RAD markers show co-segregation with unigene-EST SNP markers from the Szűcs, et al. OWB map [3]. The observed association of RAD markers with known genic-SNPs indicates they are genetically linked, suggesting some physical proximity, though the distances may be on the order of megabases. Additionally, the relative paucity of RAD markers that align to barley genes or other plant genomes indicates that only a small fraction of RAD markers originate from within coding or other conserved sequences. RAD marker development efforts from other grass species for which there is a reference genome show similar distributions of markers across coding and intergenic space [29]. When a complete barley genome sequence is available, the sequence identity and location of RAD loci will become clear. In the interim, the current availability of all barley RAD sequences is an advantage over DArTs, where only limited sequence data are publicly available.

### QTL mapping

One of the principal applications of linkage maps to crop improvement has been QTL mapping in bi-parental crosses [2]. A principal problem with many QTL mapping efforts is the limited size of the mapping population [30-33]. Recognizing that the small size of the OWB population (n = 93 and n = 82 when removing identical lines) will lead to biased estimates of QTL significance, effect, and interaction [34-37], we nonetheless proceeded with a QTL analysis of the eight traits, due to the high heritabilities (Table 2 and Table 3) and our interest in addressing two issues. The OWB population is a widely-used resource for genetic analysis and instruction: reporting the relationships of QTLs with the morphological and phenological characters segregating in the population will further develop this community resource. The RAD markers added to the map reported by Szűcs et al. [3] represent very high quality and novel data and we were interested in determining if their addition would fill gaps in the previous map and thus allow for higher resolution QTL detection.

**Table 2:** Summary of QTL based on a skeleton map derived from the 2846 loci data set.

Trait, QTL number and QTL interaction	**Chrom**.	QTL peak position	2-LOD confidence interval	Morphological and/or cloned genes within 2 LOD conf. interval	LOD	Additive effect	**R**^2*^	LOD threshold	**MIM R**^2**^	**H**^**2**^
Final leaf number								3.0	0.47	0.87
1	1H	158	(154-158)		6.9	-0.9	0.17			
2	4H	118	(117-120)	*VRN-H2*	4.6	0.7	0.10			
3	5H	157	(154-161)	*VRN-H1*	3.5	-0.7	0.09			
4	7H	29	(26-36)		3.6	0.7	0.08			
Plant height								3.1	0.82	0.94
1	1H	131	(120-136)		7.1	-6.7	0.06			
2	2H	156	(155-158)	*ZEO1*	32.1	-22.8	0.67			
3	3H	51	(43-60)		6.1	6.3	0.05			
4	6H	100	(74-104)		6.0	5.7	0.05			
Spike number								2.9	0.50	0.63
1	2H	99	(95-102)	*VRS1*	13.3	3.5	0.38			
*2*	*5H*	*43*			*2.4*	*-1.3*	*0.05*			
*3*	*6H*	*80*			*2.7*	*1.3*	*0.06*			
Spike length								3.1	0.93	0.98
1	1H	158	(152-158)		6.7	-0.5	0.00			
2	2H	156	(155-159)	*ZEO1*	45.8	-3.0	0.81			
3	3H	20	(13-23)		5.0	-0.5	0.04			
4	5H	138	(137-144)		3.8	-0.3	0.02			
5	6H	95	(93-100)		4.5	0.4	0.02			
1 × 2						0.5	0.05			
Floret number								3.0	0.87	0.97
1	1H	156	(150-158)		9.4	-5.8	0.06			
2	2H	99	(97-101)	*VRS1*	40.3	-21.6	0.79			
*3*	*3H*	*36*			*2.9*	*-2.9*	*0.01*			
4	4H	120	(112-127)	*VRN-H2*	3.6	3.3	0.02			
5	6H	91	(73-103)		4.6	3.7	0.02			
Grain number								3.0	0.74	0.92
1	1H	151	(142-156)		3.9	-4.9	0.01			
2	2H	99	(99-101)	*VRS1*	20.1	-12.3	0.46			
3	2H	156	(150-163)	*ZEO1*	10.4	-7.3	0.18			
1 × 2						2.9	0.04			
2 × 3						3.4	0.05			
Hundred grain weight								2.9	0.66	0.78
1	2H	99	(97-101)	*VRS1*	19.9	0.5	0.53			
2	6H	60	(54-61)		3.5	-0.1	0.04			
3	7H	103	(96-111)		4.2	0.2	0.05			
Grain yield								3.1	0.29	0.49
1	1H	157	(153-158)		4.4	-2.9	0.11			
2	2H	160	(150-165)	*ZEO1*	8.1	-4,1	0.23			

***Proportion of phenotypic the variance explained by individual QTL

**R^2 ^of the multi-locus model that includes QTL and their significant interactions calculated with MIM

The QTLs in italics and underlined showed a trend in the RAD-only map but did not reach the LOD threshold.

**Table 3:** Summary of QTL based on RAD-only map constructed with 436 RAD and nine morphological loci.

Trait, QTL number and QTL interaction	**Chrom**.	QTL peak position	2-LOD confidence interval	Morphological and/or cloned genes within 2 LOD conf. interval	LOD	Additive effect	**R**^2*^	LOD threshold	**MIM R**^2**^	**H**^**2**^
Final leaf number								2.8	0.41	0.87
1	1H	175	(170-175)		6.2	-0.9	0.18			
2	4H	123	(120-123)	*VRN-H2*	2.9	0.6	0.08			
3	5H	154	(148-156)	*VRN-H1*	2.8	-0.6	0.07			
*4*	*7H*	*27*			2.7	0.6	0.07			
Plant height								2.8	0.78	0.94
1	1H	144	(133-158)		6.5	-6.5	0.06			
2	2H	122	(119-128)	*ZEO1*	31.0	-21.7	0.64			
3	3H	57	(51-73)		5.6	6.0	0.05			
4	6H	112	(103-121)		5.7	5.9	0.05			
Spike number								2.7	0.56	0.63
1	2H	57	(50-64)	*VRS1*	11.4	3.6	0.40			
2	5H	9	(2-24)		3.8	-1.8	0.06			
3	6H	91	(85-98)		3.6	1.2	0.05			
1 × 2						-1.5	0.06			
Spike length								2.9	0.92	0.98
1	1H	175	(158-175)		4.9	-0.5	0.00			
2	2H	122	(120-127)	*ZEO1*	42.9	-3.1	0.82			
3	3H	20	(7-34)		5.1	-0.5	0.04			
*4*	*5H*	*105*			2.4	-0.2	0.01			
*5*	*6H*	*106*			2.7	0.3	0.02			
1 × 2						0.4	0.04			
Floret number								2.8	0.89	0.97
1	1H	175	(157-1175)		7.5	-5.3	0.05			
2	2H	57	(54-61)	*VRS1*	38.6	-21.4	0.77			
3	3H	39	(30-45)		3.2	-3.2	0.02			
4	4H	123	(117-123)		3.1	3.2	0.02			
5	6H	103	(66-120)		4.8	3.9	0.03			
Grain number								3.0	0.75	0.92
1	1H	169	(162-174)		3.8	-5.0	0.01			
2	2H	56	(53-62)	*VRS1*	18.7	-12.4	0.47			
3	2H	122	(116-130)	*ZEO1*	10.3	-7.2	0.18			
1 × 2						2.9	0.04			
2 × 3						3.3	0.05			
Hundred grain weight								2.8	0.66	0.78
1	2H	57	(54-62)	*VRS1*	19.8	0.5	0.54			
2	6H	66	(56-70)		3.5	-0.1	0.04			
3	7H	104	(86-130)		3.9	0.2	0.05			
Gain yield								2.8	0.30	0.49
1	1H	175	(170-175)		3.5	-2.8	0.11			
2	2H	122	(112-131)	*ZEO1*	6.2	-3.7	0.19			

*Proportion of phenotypic the variance explained by individual QTL

**R^2 ^of the multi-locus model that includes QTL and their significant interactions calculated with MIM

The QTLs in italic and underlined showed a trend in the full map but did not reach the LOD threshold.

As shown in Table 2, a total of 26 QTLs were found using the higher density map, with a range of one to five QTL for each individual trait. Twenty-six QTLs were also detected with the RAD-only map with a range of two to five QTL for each trait (Table 3). Twenty-three QTLs were significant and detected in both maps. Of the three QTL that were significant in the full map, but not the RAD-only map, all showed a trend in the RAD-only map but did not reach the LOD threshold. Three QTL significant in the RAD-only map but not in the full map showed a trend in the full map but did not reach the LOD threshold. Therefore, RADs alone, or in combination with other markers, are suitable for QTL mapping. This supports the quality of the RAD data, since a key issue for QTL detection is marker quality, given adequate genome coverage [37].

The following results highlight findings from the higher density skeleton map (Table 2), based on the assumption that by providing the most thorough coverage it optimizes QTL estimates. However, the same large-effect QTL were detected with the RAD-only map (Table 3). As shown in Table 2, eleven of the twenty-six QTL were associated with four genes: *ZEO-1*, *VRS-1*, *VRN-H1 *and *VRN-H2*, and the largest effect QTL for all traits were associated with *ZEO-1*and/or *VRS-1*. The favorable alleles for height, spike length, grain number and grain yield came from the OWB recessive parent (normal height, long spike, and six-row) at *ZEO-1*. The OWB recessive parent also contributed favorable alleles for floret and grain number at *VRS-1*. At this locus, the OWB dominant parent (dwarf height, short spike, and two-row) contributed favorable alleles for spike number and hundred grain weight. Although *VRS-1 *and *ZEO-1 *were both coincident with yield component QTL, only *ZEO-1 *had a significant effect on grain yield. This is probably due to yield component compensation associated with *VRS-1 *and negative pleiotropic effects of the *ZEO-1 *dwarf allele. This extreme dwarfing allele will not be as immediately useful to agriculture as the *Rth-B1 *and *Rht-D1 *genes of wheat [38]. Interestingly, QTLs for final leaf number were coincident with *VRN-H1 *and *VRN-H2*. These two genes interact epistatically to determine vernalization sensitivity [39]. The OWB dominant and recessive parents, respectively, have dominant (winter) and spring (recessive) alleles at *VRN-H2 *allele. Therefore, it is of interest that the OWB dominant allele at *VRN-H2 *is associated with higher final leaf number, even though there is no binding site in *Vrn-H1 *for the repressor encoded by *VRN-H2 *since both parents have the same recessive (spring) allele at *VRN-H1 *[40]. The higher final leaf number QTL allele coincident with *VRN-H1 *may be a consequence of regulation of other regions in *VRN-H1 *besides *VRN-H2*. There were epistatic QTL interactions for spike length, and grain number but these effects were very small in comparison to the main effects. The QTL we report for the OWB population can be aligned with QTL for other traits assessed in other germplasm via the GrainGenes QTL summary http://wheat.pw.usda.gov/ggpages/maps/OWB/.

## Conclusions

In this study we showed that sequenced RAD markers were sufficient to generate a high quality linkage map comparable to current OWB SNP-based maps. The success of linkage map construction supports the reliability of the sequenced RAD markers based on the following criteria i) a small number of singletons ii) consistency with non-RAD marker order iii) segregation distortion between maps in equivalent positions iv) comparable genome coverage and v) comparable map lengths. Construction of this linkage map could serve as a bridge to allow identification of loci associated with traits of interest, thus facilitating gene discovery and manipulation. The consistency of QTL results between RAD and RAD + prior marker maps confirms that sequenced RAD markers will be useful for developing genetic maps and QTL tagging. Therefore, sequenced RAD markers can contribute to the enrichment of molecular marker resources and have useful applications in molecular breeding.

Ongoing optimization of the RAD marker system will foster more sophisticated analysis in future studies. Selection of nucleases that generate more markers will allow higher density linkage maps to be constructed, while improvements in sequencing chemistries and fragment preparation protocols will permit longer read lengths for comparative genome analysis. Additionally, sequenced RAD markers arrayed in genetic maps would be of significant benefit as a scaffold framework for placement of shotgun sequence reads and *de novo *genome assembly refinement.

## Methods

### Plant material

The mapping population consists of 93 doubled haploid (DH) lines. The DH lines were produced from the F1 of the cross of the Wolfe recessive and dominant marker stocks using the *Hordeum bulbosum *method [19]. In the course of this research we determined that nine sets of DH lines had identical genotypes. Specifically, the following sets of lines are identical: set1 = DH 1,4,27,62; set2 = DH 16,71; set3 = DH 5,18; set4 = DH 31,58; set5 = 35,50; set6 = DH 15, 47 set7 = DH 61, 88; set8 = DH 22,70; set9 = DH 80,77. Retention of one genotype per set (DH 4, 16, 18, 31, 35, 47, 61, 70 and 77) reduces the population size to 82. This report describes mapping and QTL analysis using the OWB population of 82 lines. In order to ascertain the bias introduced by duplicate lines (an unintended consequence of the DH production process), all analyses were also conducted with a population size of n = 93 [20]. Genomic DNA was extracted from young leaf tissue of a single plant representing each DH line, and each of the parents, using DNeasy plant maxi kits (QIAGEN Inc. California, USA).

### RAD protocols

OWB genomic DNA from the selected mapping population was digested with the restriction endonuclease *SbfI *and processed into RAD libraries similarly to the method of Baird et al. [14]. Briefly, P0 (parental genotypes) and DH (progeny) genomic DNA (~300 ng; from each sample) was digested for 60 min at 37°C in a 50 μL reaction with 20 units (U) of *SbfI *(New England Biolabs [NEB]). Samples were heat-inactivated for 20 min at 65°C. 2.0 μL of 100 nM P1 Adapter(s), a modified Solexa^© ^adapter (2006 Illumina, Inc., all rights reserved). *SbfI *P1 adapters each contained a unique multiplex sequence index (barcode) which is read during the first four nucleotides of the Illumina sequence read. 100 P1 nM adaptor were added to each sample along with 1 μL of 10 mM rATP (Promega), 1 μL 10× NEB Buffer 4, 1.0 μL (1000 U) T4 DNA Ligase (high concentration, Enzymatics, Inc), 5 μL H_2_O and incubated at room temperature (RT) for 20 min. Samples were again heat-inactivated for 20 min at 65°C, pooled and randomly sheared with a Bioruptor (Diagenode) to an average size of 500 bp. Samples were then run out on a 1.5% agarose (Sigma), 0.5× TBE gel and DNA 300 bp to 700 bp was isolated using a MinElute Gel Extraction Kit (Qiagen). End blunting enzymes (Enzymatics, Inc) were then used to polish the ends of the DNA. Samples were then purified using a Minelute column (Qiagen) and 15 U of Klenow exo^- ^(Enzymatics) was used to add adenine (Fermentas) overhangs on the 3' end of the DNA at 37°C. After subsequent purification, 1 μL of 10 μM P2 adapter, a divergent modified Solexa^© ^adapter (2006 Illumina, Inc., all rights reserved), was ligated to the obtained DNA fragments at 18°C. Samples were again purified and eluted in 50 μL. The eluate was quantified using a Qubit fluorimeter and 20 ng of this product was used in a PCR amplification with 20 μL Phusion Master Mix (NEB), 5 μL of 10 μM modified Solexa^© ^Amplification primer mix (2006 Illumina, Inc., all rights reserved) and up to 100 μL H_2_O. Phusion PCR settings followed product guidelines (NEB) for a total of 18 cycles. Samples were gel purified, excising DNA 300-650 bp, and diluted to 1 nM.

To promote SNP identification in low-copy, gene-rich regions of the barley genome, a species with ~90% retroelement content, selection of a restriction enzyme that does not fragment repetitive-class DNA is desirable. Previous studies have documented epigenetic modification of CpG, CpNpG and CpNpN nucleotides with 5-methylcytosine (5 mC) in retroelement-dense regions of many plant genomes, including triticale [41-43]. Methylation-sensitive type II restriction endonucleases, which do not cleave 5 mC-modified DNA, can be used to specifically sample the hypomethylated genomic fraction and are commonly used in other restriction-enzyme based genetic marker systems [44]. We selected the restriction enzyme *Sbf*I, (5'CCTGCA/GG'3) with a recognition site containing two CpNpG trinucelotide repeats for RAD sequencing of the barley genome.

### Illumina Sequencing

The constructed OWB libraries were run on an Illumina Genome Analyzer II at the University of Oregon High Throughput Sequencing Facility. Illumina/Solexa protocols were followed for single read (1 × 36 bp) sequencing chemistry. A total of 20.4 M Illumina reads were obtained from sequencing of the population. Sequences are available at the Sequence Read Archive http://www.ncbi.nlm.nih.gov/Traces/sra/, at accession SRA020593.

### Sequence Analysis and SNP Discovery and Genotyping

Internal Floragenex sequence tools and custom PerlScripts were used for processing of raw Illumina/Solexa data. Data from multiple Illumina/Solexa sequence channels was segregated by the appropriate four nucleotide multiplex identifier (MID) assigned to each sample. All reads were trimmed to 28 nucleotides from the 3' end of genomic sequence to avoid using bases with a high Illumina sequence error rate.

### Sequence Alignment and Comparative Genomics

The short-read alignment program Bowtie [21] was used for mapping of polymorphic barley RAD sequence loci (Additional file 4: Table S4) to the comprehensive *Hordeum *gene index (HvGI v10.2) database from the Dana-Farber Cancer Institute [22]. Both tentative consensus (TC) and singleton expressed sequence tags (ESTs) were used in analysis. Briefly, sequences corresponding to all 530 polymorphic RAD loci were aligned against the HvGI assembly. Two criteria were imposed for sequence mapping. First, a maximum of three nucleotide mismatches and no gaps between the RAD sequence and reference were permitted for any alignment. Second, each sequence had to anchor to a single unique position to be scored. For macro-scale syntenic mapping of barley RAD sequences to other grass genomes, we extended the CIP/CALP (Conserved Identity Percentage/Conserved Alignment Percentage) method previously used in Triticale comparative analysis [26]. 30 bp RAD sequences ordered by the linkage map were aligned against the *Oryza sativa *and *Brachypodium distachion *chromosome assemblies using relaxed Bowtie alignment parameters. Bowtie is able to tolerate up to three nucleotide mismatches between query and reference, translating to minimum values of 90% and 90% respectively for CIP and CALP.

### Linkage mapping

Two linkage maps were constructed. The first map was built with only the RAD data and data for nine morphological markers (Table 4). The morphological marker data were reported by Szűcs et al. [3] and were included because they provide anchors for equating linkage groups with six of the seven barley chromosomes. A second map was built using RAD data and all 2383 data points reported by Szűcs et al. [3]. Each linkage map was constructed using JoinMap 4 [17]. Linkage groups were identified using minimum LOD values of 5. The Monte Carlo Maximum Likelihood (ML) mapping algorithm was used to determine the orders of markers within each linkage group. Map distances were calculated using the Haldane's mapping function. Maps were drawn using MapChart v2.2 [45]. Data used for linkage map construction are available at Oregon Wolfe Barley Data and GrainGenes Tools http://wheat.pw.usda.gov/ggpages/maps/OWB/.

**Table 4:** Anchor markers for RAD-only map construction.

Locus	Gene	Chromosome	Phenotype	Gene
*VRS-1*	*HvHox1*	2H	Two-row inflorescence (*Vrs1Vrs1*)/six-row inflorescence (*vrs1vrs1*)	[49] GenBank:[AB489122.1]
*ZEO-1*	NA	2H	Dwarf plant with compact head (*Zeo1*)/normal height and head length (*zeo1*)	
*ALM*	NA	3H	Green lemma and nodes (*Alm*)/albino lemma and nodes (*alm*)	
*HSH*	NA	4H	Hairs on lower leaf sheaths (*Hsh*)/lack of hair on lower leaf sheaths (*hsh*)	
*SRH*	NA	5H	Long hairs on rachilla (*Srh*/short hairs on rachilla (*srh*)	
*ROB*	NA	6H	Green lemma and nodes (*Rob*)/orange lemma and nodes (*rob*)	
*WX*	*GBSS-I*	7H	Wild type endosperm starch (*Wx*)/waxy endosperm starch (*wx*)	[50] GenBank:[AF486518.1]
*NUD*	NA	7H	Hulled seed (*Nud*)/hulless seed (*nud*)	[51] GenBank:[AP009567]
*LKS2*	NA	7H	Long awn (*Lks2*)/short awn (*lks2*)	

Nine morphological marker loci used for linkage map construction, together with RAD loci, in the Oregon Wolfe Barley mapping population, showing chromosome assignments, phenotypes, and genes (if known).

### Phenotyping

In order to assess the utility of the RAD and RAD + SNP map for quantitative trait locus (QTL) detection, data on phenological and reproductive fitness phenotypes were obtained for the 93 DH lines and the two parents. Individual plants were grown in 13.5 cm pots at the Oregon State University greenhouses (Corvallis, Oregon USA). Supplemental light was used to maintain a 16 h light/24 h photoperiod. Temperatures were maintained at a constant 18 ± 2°C day and night temperature. Each DH and parental line was replicated twice. Eight traits were measured on each plant. The trait abbreviations and definitions are as follows: (1) *Final leaf number *(FLN) was recorded as the total number of leaves on the main stem of each plant; (2) *Plant height *(PH) was measured as the distance (in cm) from the soil surface to the tip of the tallest inflorescence (spike), exclusive of awns, if present; (3) *Spike number *(SN) was the actual count of the total number of fertile spike on each plant. Three stems with fertile spikes were selected at random from each plant for determining the following traits, and the individual values were averaged: (4) *Spike length *(SL) was measured as the length (in cm) from the first rachis internode to the top of the final fully formed floret, exclusive of awn; (5) *Floret number *(FS) was the count of the number of florets (fertile and sterile) per spike; (6) *Grain number *(GN) was the count of the number of seed-containing florets per spike; (8) *Hundred grain weight *(HGW) was the weight (in g) of 100 grains. Grain yield per plant (GY) was estimated by the function GY = SN*GN*HGW. Phenotype data are available at Oregon Wolfe Barley Data and GrainGenes Tools http://wheat.pw.usda.gov/ggpages/maps/OWB/.

### QTL analysis

QTL analyses were performed for each of the nine traits using the RAD-only and RAD + 2383 locus maps as follows: For the RAD-only map, all data included in the linkage map were used. For the RAD + 2383 locus map, a skeleton map was developed using a single marker (selected at random) for an average marker density of 2 cM and a total of 624 markers. The QTL analyses were conducted with QTL Cartographer Version 2.5 [46] using Composite Interval Mapping (CIM) [47]. Up to seven cofactors for CIM were chosen, using a forward-selection backward-elimination stepwise regression procedure with a significance threshold of 0.1. The walk speed was set to 1 cM, and the scan window to 50 cM beyond the markers flanking the interval tested. Experiment-wise significance (α = 0.05) likelihood ratio test (LR) thresholds for QTL identification were determined with 1,000 permutations, and expressed as LOD (LOD = 0.217 LR). Epistatic interactions between QTL were evaluated with the Multiple Interval Mapping (MIM) [48] method implemented in Windows QTL Cartographer using Bayesian Information Criteria (BIC-M0). Broad-sense heritability values were estimated using the following formula:

$$H^{2}=\frac{\sigma_{G}^{2}}{\sigma_{G}^{2}+\frac{\sigma_{e}^{2}}{r}}$$

where $\sigma_{G}^{2}$ represent the genetic variance, $\sigma_{e}^{2}$ the residual variance and r the number of replicates per genotype.

## List of abbreviations

DArT: Diversity Array Technology; DH: Doubled haploid; EST: Expressed Sequence Tag; GBS: Genotyping by-sequencing; LOD: Logarithm of odds; OWB; Oregon Wolfe Barley; QTL: Quantitative Trait Locus; RAD: Restriction site Associated DNA; SNP: Single Nucleotide Polymorphism;

## Competing interests

RWN is an employee of, and EJ a shareholder in, Floragenex - an organization which offers commercial RAD sequencing services. This organization is not financing the manuscript.

## Authors' contributions

EAJ handled raw sequence analysis and developed PerlScripts for barley sequence genotyping. RWN drafted portions of the manuscript and performed alignments and comparative genomic analysis of the short read data. PMH drafted portions of the manuscript. AC-M drafted portions of the manuscript and performed linkage and QTL mapping analyses. CY performed linkage and QTL analyses, and assembled the final draft. LC, AC, TF assisted in generating data and provided insightful suggestions for analysis and interpretation. All authors have read and approved the final version of this manuscript.

## Supplementary Material

Additional file 1 **Table S1: Oregon Wolf Barley DH Sequencing Summary**. The aggregate sequence reads obtained for both parents and each member of the OWB mapping population are provided. Sequencing coverage for each sample is also calculated based on the formula (Number of SbfI genome sequences from Barley genome/raw sequences obtained). Clustering of RAD data from multiple individuals indicates there are approximately 10,000 SbfI sequences in the typical *Hordeum *genome. Click here for file

Additional file 2 **Table S2: Oregon Wolf Barley DH RAD Marker Sequences**. The sequence data for each RAD marker positioned on the genetic map is provided in this spreadsheet. Click here for file

Additional file 3 **Figure S1: Linkage map of Oregon Wolfe Barley population based on RAD markers**. Click here for file

Additional file 4 **Table S4: Oregon Wolf Barley RAD EST/Genome Alignments**. Bowtie alignments of OWB RAD markers to three sequence databases are provided: The *Hordeum *gene index (HvGI v10.2) from the Dana-Farber Cancer Institute, the MSU Rice Genome Annotation Project Release 6.0 (January 30, 2009) and the 8× *Brachypodium *Genome Assembly from brachypodium.org. The table columns detail, from left to right: the OWB marker name, sequence alignment orientation, the name (either EST/contig/chromosome identifier) and position (in bp) of the sequence alignment within the reference assembly, the sequence of the RAD marker and any variations observed between query (RAD marker) and reference. Variations are reported as: position in read, reference allele and query allele. Click here for file

Additional file 5 **Table S5: Syntenic Oregon Wolf Barley/Brachypodium RAD Marker Sequences**. Bowtie alignments of OWB RAD markers to the 8× *Brachypodium *Genome Assembly from brachypodium.org are shown. OWB RAD markers have been ordered by linkage group and map position. The corresponding alignment positions for each marker on the Bd21 assembly are shown in columns at right, with chromosome, alignment position, sequence and observed sequence variation between *Brachypodium *and OWB RAD markers. Click here for file
