# Supplementary figures and images for: Construction and application for QTL analysis of a Restriction Site Associated DNA (RAD) linkage map in barley
Source: BMC Genomics. 2011 Jan 4;12:4. doi: 10.1186/1471-2164-12-4 (PMC3023751; doi:10.1186/1471-2164-12-4)

# Additional file 3: Figure S1 Linkage map of Oregon Wolfe Barley population based on RAD markers

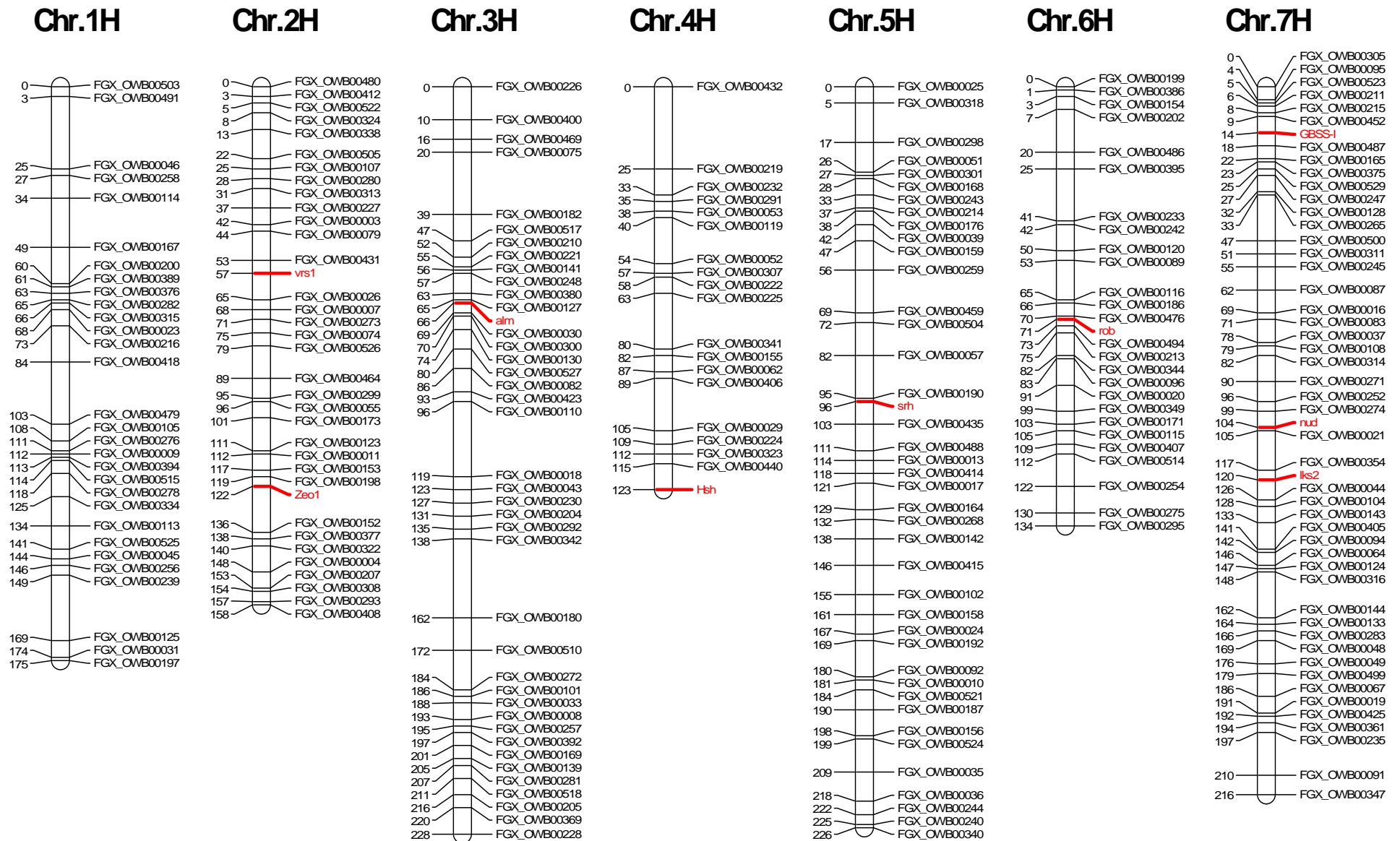

Supplement: Additional file 3 — Figure S1: Linkage map of Oregon Wolfe Barley population based on RAD markers. [file 1471-2164-12-4-S3.PDF]
